# Hebbian Control of Fixations in a Dyslexic Reader: A Case Report

**DOI:** 10.3390/brainsci13101478

**Published:** 2023-10-19

**Authors:** Albert Le Floch, Guy Ropars

**Affiliations:** Unité de Formation et de Recherche Sciences et Propriétés de la Matière, University of Rennes, Campus de Beaulieu, Building 7, Laboratory P938, 35042 Rennes Cedex, France; albert.lefloch@laposte.net

**Keywords:** eye fixations, dyslexia, Hebb mechanisms, reading, eye tracking

## Abstract

When reading, dyslexic readers exhibit more and longer fixations than normal readers. However, there is no significant difference when dyslexic and control readers perform only visual tasks on a string of letters, showing the importance of cognitive processes in reading. This linguistic and cognitive processing requirement in reading is often perturbed for dyslexic readers by perceived additional letters and word mirror images superposed on the primary images on the primary cortex, inducing internal visual crowding. Here, we show that while for a normal reader, the number and the duration of fixations remain invariant whatever the nature of the lighting, the excess of fixations and total duration of reading can be controlled for a dyslexic reader using the Hebbian mechanisms to erase extra images in optimized pulse-width lighting. In this case, the number of fixations can then be reduced by a factor of about 1.8, recovering the normal reading experiment.

## 1. Introduction

Fixational eye movements are a fundamental aspect of vision [1,2,3]. Even when a compound eye like that of drosophila is rigidly attached to the skeleton, muscles have recently been shown to move the retina itself [4]. Anomalous eye movements have been discussed for some time now, in particular for children and adults with dyslexia [2,5] who lag behind normal readers on embedded letters and on words [6]. Binocular coordination of saccades [2,7] and vergence anomaly [8] have also been observed. Moreover, many studies have found that an excessive number of overly long fixations generally perturb dyslexics in reading but not in visual tasks [9,10,11]. Similar results have also been observed in languages with a higher grapheme–phoneme correspondence, such as German [12,13,14], and even in logographic languages with a deep orthography, such as Chinese [15]. Indeed, reading makes additional demands with respect to linguistic and cognitive processing. However, the saccadic patterns observed in readers with dyslexia seem to be the result and not the cause of their reading disabilities [2,16].

This excess of fixations in dyslexia, which has been studied by many groups [2,5,10,17,18,19,20,21,22] using different currently available eye-tracking systems [23], seems to provide a useful guide for detecting and predicting dyslexia using machine learning based on the eye-tracking technique [24,25,26,27,28,29,30,31]. The role of external crowding [32,33] has been studied and discussed in developmental dyslexia [34,35]. Various remediation methods, including increasing letter spacing [36,37], the use of colour filters [38,39], and e-reading with spaced letters [40], have been shown to help people with dyslexia.

A possible role for the lack of asymmetry between the Maxwell centroids in dyslexia inducing an absence of ocular dominance and the frequent existence of perceived extra mirror or duplicated images has also been proposed [41]. The associated internal visual crowding due to callosal interhemispheric projections of letters and words can perturb brain connectivity [42], particularly in the reading process. The aim of this paper is to show the role of this internal visual crowding in eye movements, especially in eye fixations during reading. With a higher number of fixations being an undisputed symptom of dyslexia, it is tempting to try to control them using the Hebbian mechanisms [43] at the synapses of the primary cortex. Taking into account the small delay associated with the interhemispheric transfer through the corpus callosum, the mirror images can be weakened by pulsed stimuli during silent reading.

## 2. Methods

To investigate this possibility, we have electronically modified a computer screen equipped with an eye tracker to optimize the lighting regime and hence control the internal visual crowding. The presence or absence of this internal visual crowding could then worsen or improve the fixational movements, suggesting a causal relationship [44] with the reading deficits.

### 2.1. Participants

We tested two male undergraduate students (21 years old) following the same physics courses in the third year of a Bachelor’s degree at the University of Rennes. The two students were native French speakers with normal vision. Neither of them had been diagnosed with a psychiatric disorder or any other form of cognitive disorder. The student with dyslexia and the student with normal reading characteristics were aware of the purpose of the study and gave informed written consent before participating. The entire investigation was conducted according to the principles expressed in the Declaration of Helsinki.

### 2.2. Foveascope

**Figure 1:**
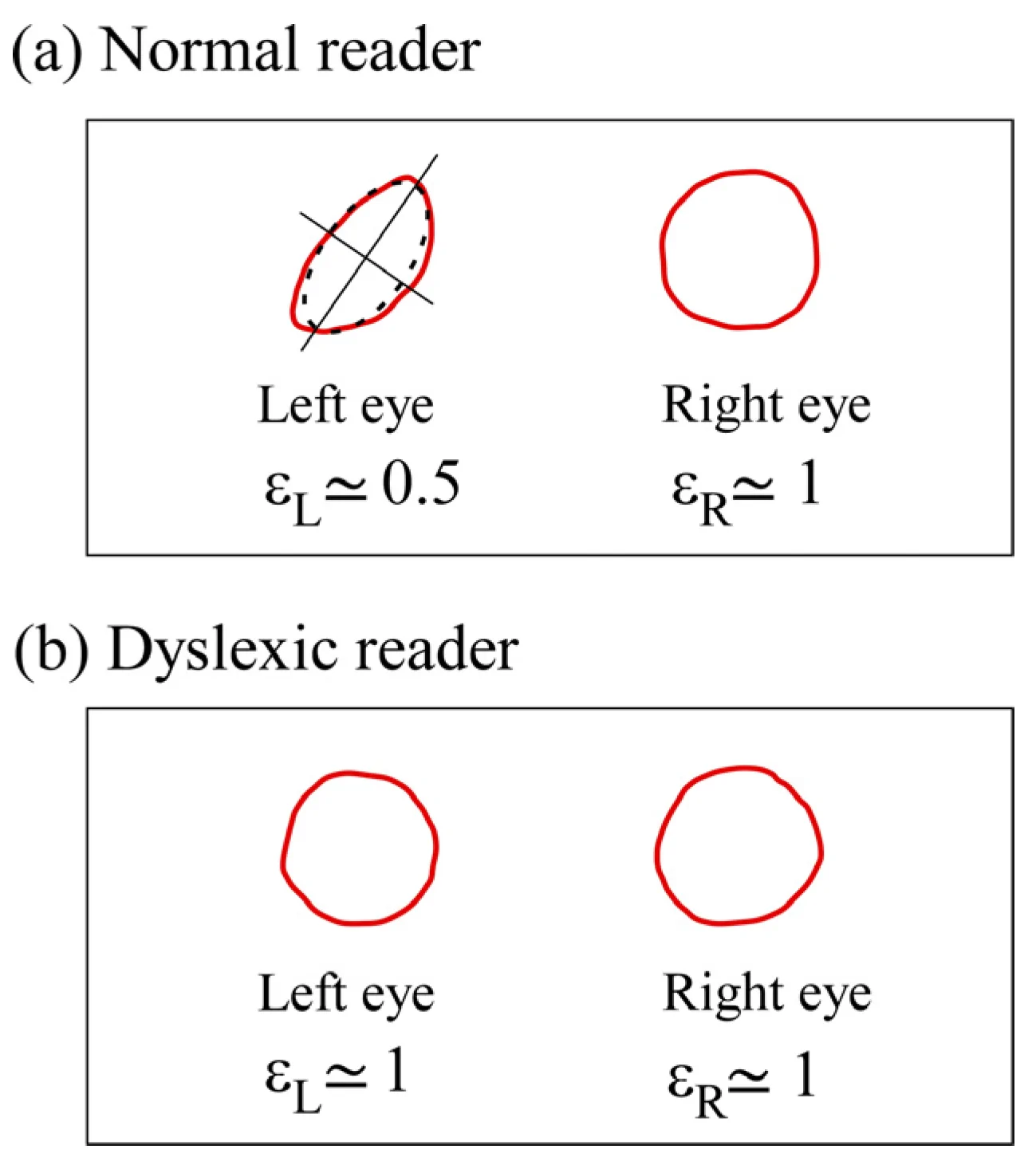
Maxwell’s centroid profiles. (**a**) For a normal reader. (**b**) For a dyslexic reader. The ellipticity ε is defined by the ratio of the lengths of the two axes of the osculating ellipse (dotted line). The profiles show the asymmetry for the normal reader with an ellipticity difference between the two eyes $∆\varepsilon =\varepsilon_{R}-\varepsilon_{L}≃0.5$ and the lack of asymmetry for the dyslexic reader with $∆\varepsilon =\varepsilon_{R}-\varepsilon_{L}≃0$ . The corresponding mean diameters on the retinas are between 100 to 150 µm.

The setup described in [41] was adopted to investigate the two Maxwell centroid profiles, i.e., the blue cone-free areas at the centre of the foveas, and to record their asymmetry (Figure 1). The contrast of the Maxwell centroid entoptic image is optimized by using a blue-green exchange filter. Each observer adjusts the modulation frequency around 0.2 Hz to suit his own vision.

### 2.3. Noise-Activated Negative Afterimages

**Figure 2:**
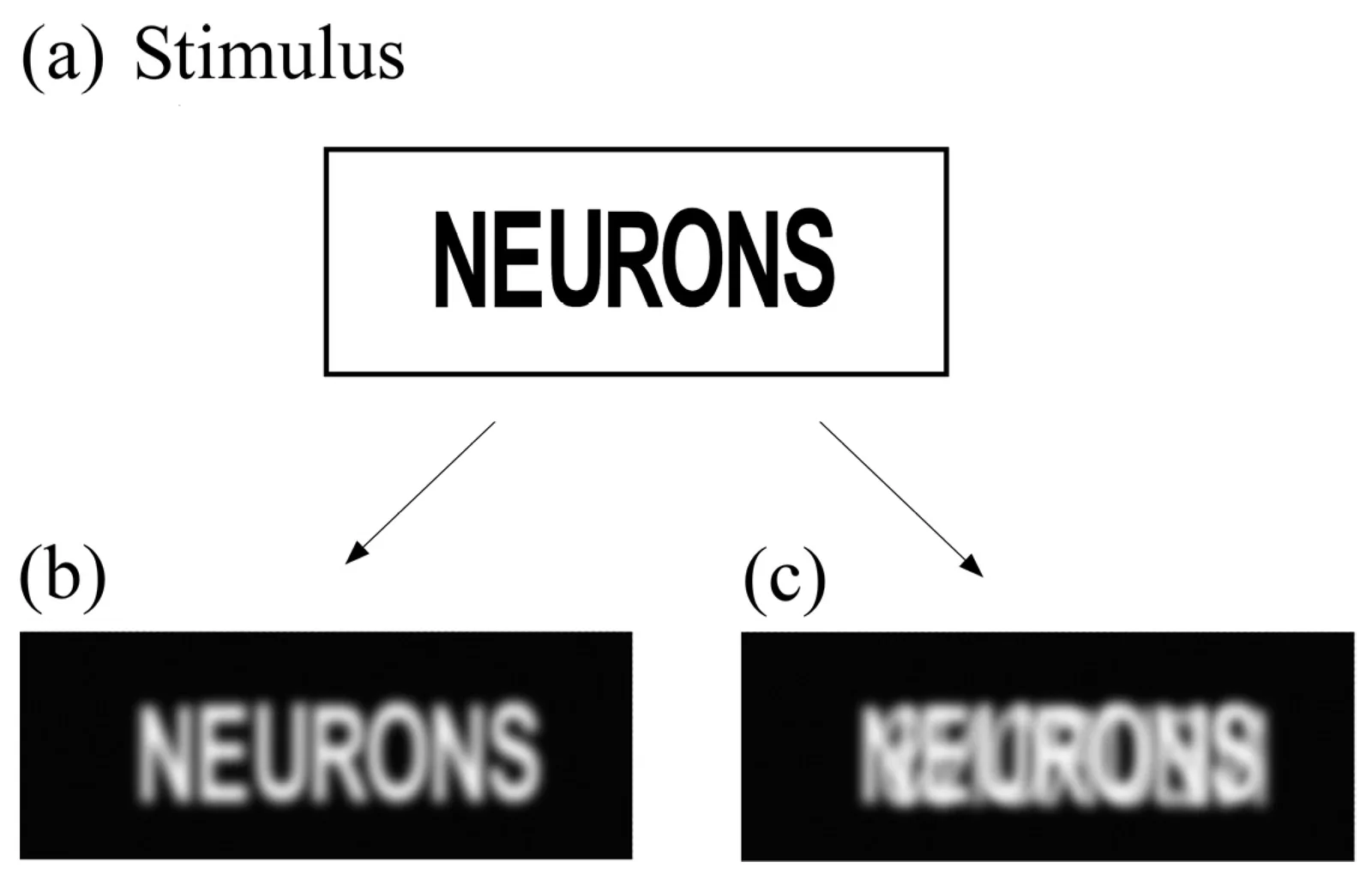
Noise-activated negative afterimages. The negative afterimages are perceived when the diffuse noise through the eyelids falls on the retina. The negative afterimages are then reconstructed in each case. (**a**) Stimulus: NEURONS. (**b**) Noise-activated negative afterimage perceived by the normal reader. (**c**) Noise-activated negative afterimage perceived by the dyslexic reader. The mirror image induces internal visual crowding.

Retinal neurons are non-linear and bistable; therefore, they are sensitive to noise [45]. In general, closed eyelids allow 2% of incident light to pass through. This diffuse light leads to noise falling on the retina, which can activate the retinal cells and the primary images arriving on layer 4 of the primary cortex, which is the only layer that is sensitive to diffuse light [46] and receives most of the signals from the retinas. After fixating for a few seconds on a stimulus (Figure 2a), such as the word “NEURONS” placed on a window illuminated by daylight, closing their eyes, blocking out all light with by placing their hands over the eyes, and then shifting them periodically apart, the observer perceives the negative afterimage of the stimulus, as shown in Figure 2b for the normal reader and in Figure 2c for the dyslexic reader.

### 2.4. Eye Tracking Movements with the Stimuli Provided by an Electronically Modified Computer Screen

**Figure 3:**
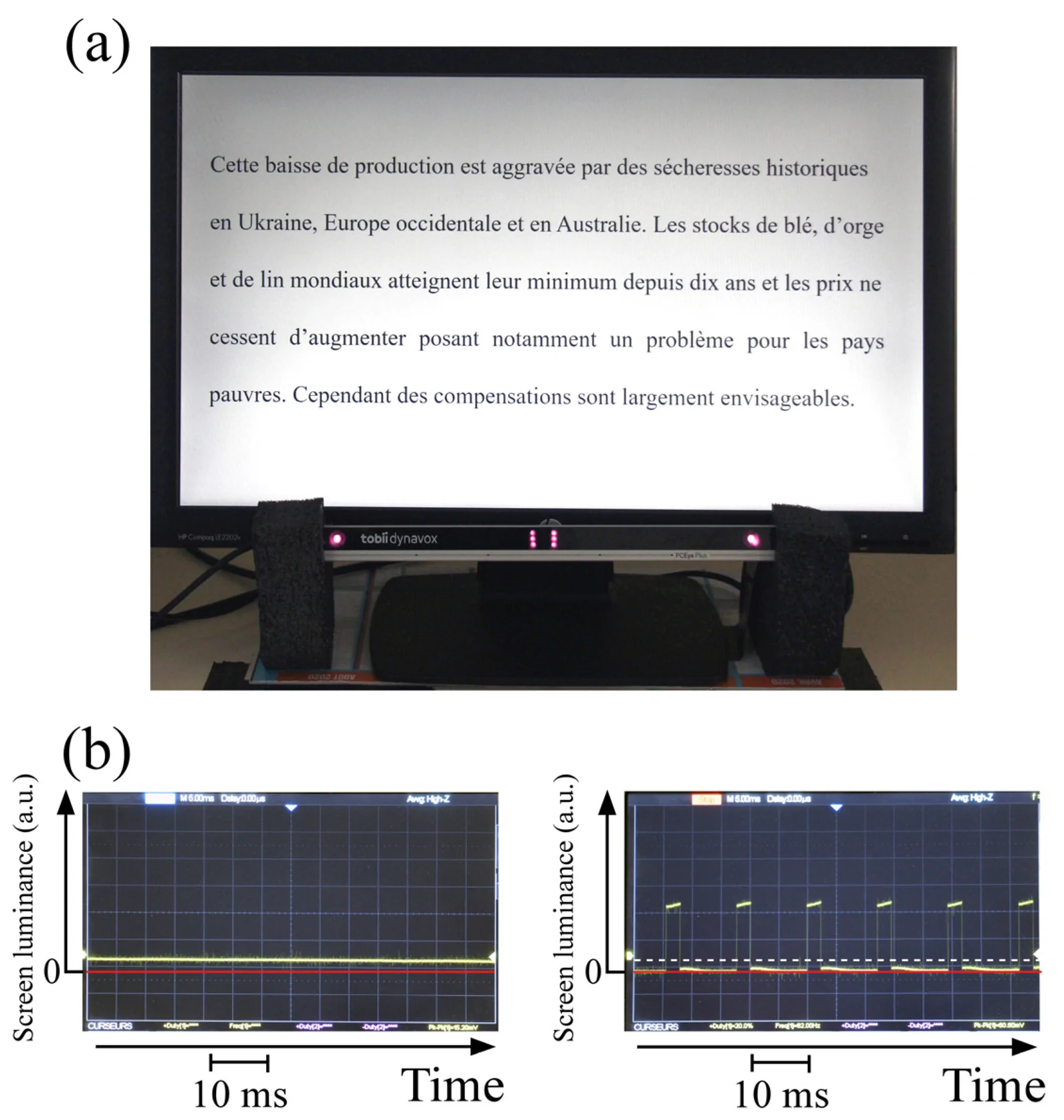
(**a**) The eye tracker system with an electronically modified screen. (**b**) Screen luminance versus time in the continuous regime (left side) and in the pulsed regime (right side). The mean illuminance (dotted line) is exactly the same as in the continuous regime.

The computer screen (Hewlett Packard Compaq LE 2202x, Bloeblingen, Germany) was electronically modified so as to work in the continuous lighting regime (CW) or in a pulse-width modulated regime with a variable frequency from 60 to 120 Hz. The optimal frequency for the dyslexic reader was obtained by continuously varying the frequency between 60 Hz and 120 Hz, and it corresponds to a delay of about 10 ms for the interhemispheric transfer through the corpus callosum. The dyslexic reader looks at the screen while the frequency is varied and is able to optimize for himself the frequency at which disturbing secondary mirror images disappear. The eye tracker used is a commercial infrared system (Tobii dynavox PCEye Plus, Version 1.3) with a sampling frequency of 60 Hz that is used with software (Tobii Dynavox Gaze Viewer, V.1.2.0.63881) for rendering data as images and movies with gaze plots. The eye tracking analysis algorithm provides the total number of fixations and the total reading time. Calibration was carried out using a 9-point routine. Participants were seated about 60 cm from the screen, which is within the optimal range for recording, as described in the eye tracker manual. A chin and forehead rest was used in order to limit head movement. The experiment was carried out in a dark room. Figure 3a shows the whole system with the corresponding screen luminance versus time recorded in the continuous (left side of Figure 3b) and pulsed regimes (right side of Figure 3b) using an N-type Si ultrafast FND 100 photodiode. The mean luminance was the same in both regimes and was measured using a lux meter (Roline model Ro 1332, Taiwan). In the pulsed regime, the cyclic ratio can be adjusted continuously. We used a French text adapted for the assessment of dyslexia in adults [47]. We divided this into four parts of similar length and difficulty. These four texts were presented successively on the computer screen and were written in black Times New Roman font on a white background. Each text started with a capital letter and was 5 lines and approximately 291–299 characters (51.8 ± 2.4 words) long. The mean character width was about 0.45°. The participants were asked to read the texts silently.

## 3. Results

### 3.1. The Asymmetry of Maxwell’s Centroids

The two students recorded the profiles of their two Maxwell’s centroids, as shown in Figure 1. The ellipticity of each profile $\varepsilon_{R}$ and $\varepsilon_{L}$ for the right and left eye, respectively, is measured using the osculating ellipse. The asymmetry is defined by $∆\varepsilon =\varepsilon_{R}-\varepsilon_{L}$ . For the normal reader, the asymmetry was ∆ε ≃0.5 , with a quasi-circular profile in the right eye, which is thus his dominant eye (Figure 1a). In contrast [41], for the dyslexic reader, the two profiles are similar (Figure 1b) and quasi-circular ( $\varepsilon_{R}≃\;\varepsilon_{L}$ ≃1), and the lack of asymmetry induces an absence of ocular dominance and an internal visual crowding (Figure 2b). Note that for a normal reader, when the blue cone topographies are different in the two foveas, the green and red cone topographies are also automatically slightly perturbed. The asymmetry induces two slightly different retinal images and ocular dominance, as well as two slightly encoded different retinoptic maps on layer 4 of the primary cortex, where virtually all signals from the retinas arrive [48,49].

### 3.2. Internal Visual Crowding

After a binocular fixation on a stimulus such as NEURONS (Figure 2a), the normal reader perceived only the primary negative afterimage (Figure 2b), whereas the dyslexic reader with mirror images perceived the superposition of the primary and mirror images as in Figure 2c. Although the mirror image is weaker, confusion of letters is possible, and syllables are difficult to decipher. In another study, mirror images corresponding to symmetric projections between the two hemispheres were observed in 60% of a cohort of 160 dyslexic children, whereas duplicated images corresponding to non-symmetric projections were observed in 35% of the children [50]. As noted previously [41], small lateral shifts in the projected images generally occur, leading to different levels of severity of internal visual crowding.

### 3.3. Fixations during Reading

**Figure 4:**
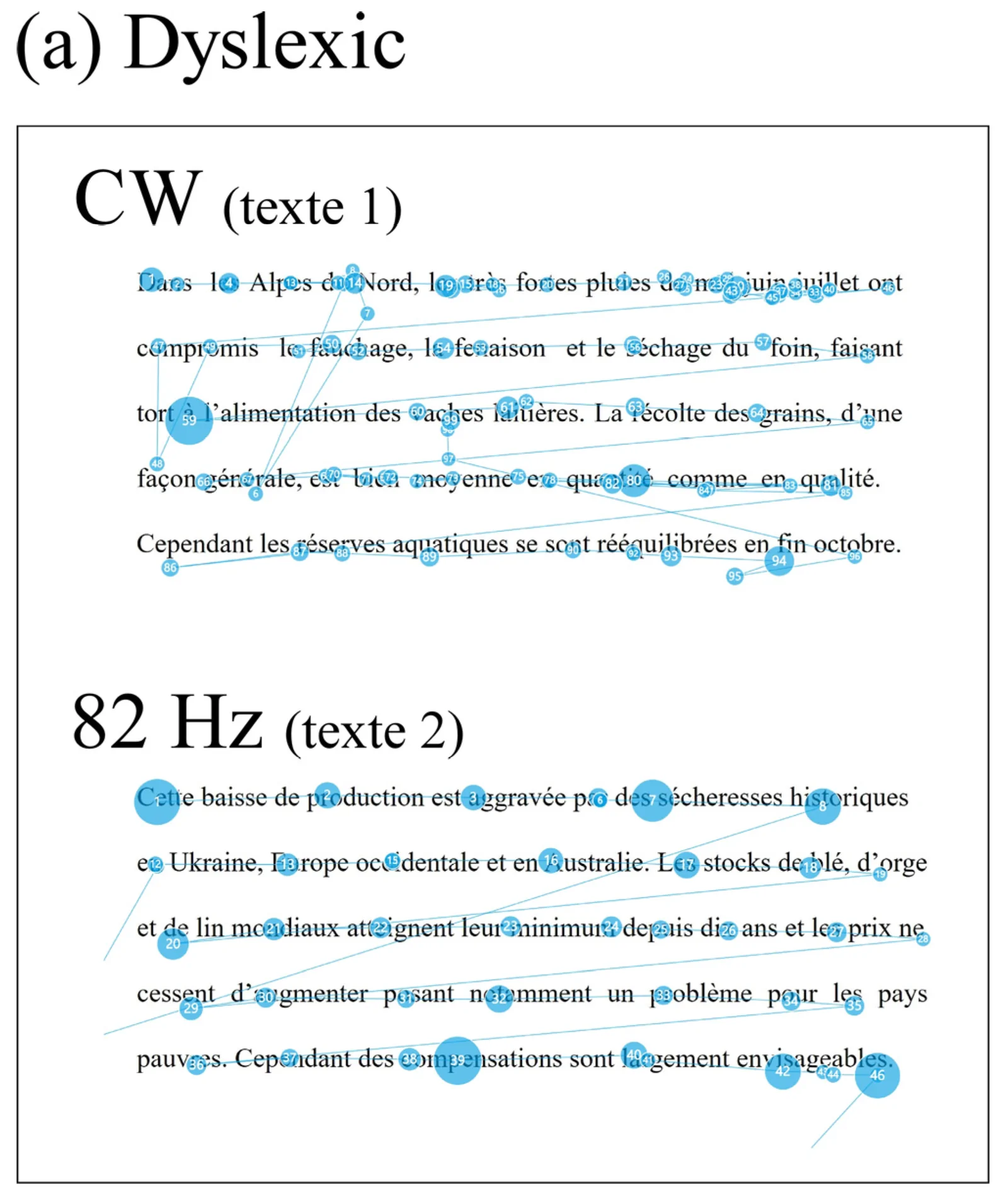

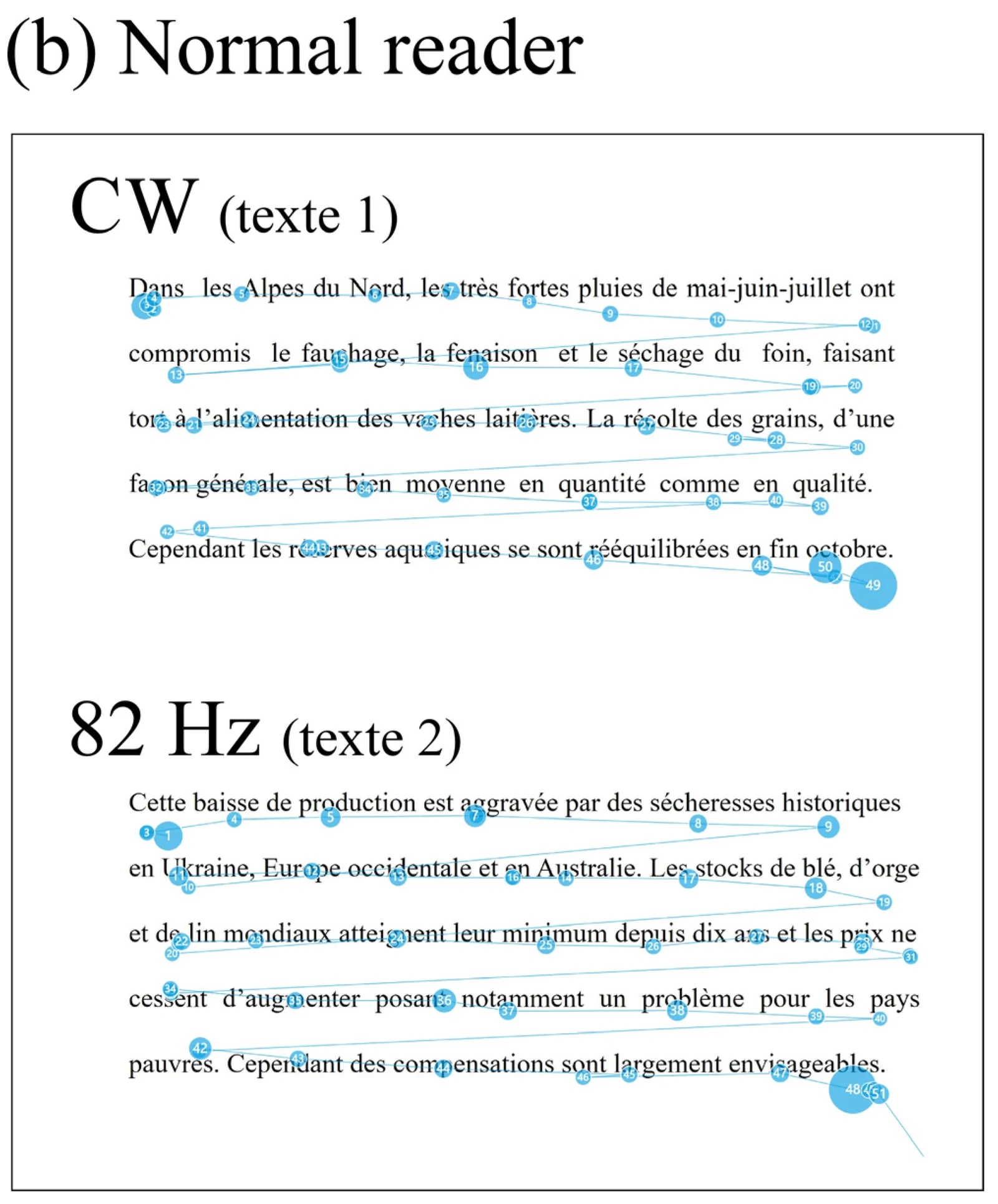
Eye movement patterns during reading. (**a**) Results for the dyslexic reader in the continuous (top) and pulsed regime (82 Hz—bottom). (**b**) Results for the control reader in the continuous (top) and pulsed regime (82 Hz—bottom).

The eye movement patterns during reading are shown in Figure 4 for the two readers under the continuous wave (CW) and pulsed light regime for two texts. While for the normal reader, 50 fixations are necessary independently of the light regime (Figure 4b), for the dyslexic reader, 95 fixations are necessary in the usual continuous regime (top of Figure 4a); however, for the latter, only 46 are necessary in the optimized pulsed light regime at 82 Hz (bottom of Figure 4a), similar to the normal reader.

**Figure 5:**
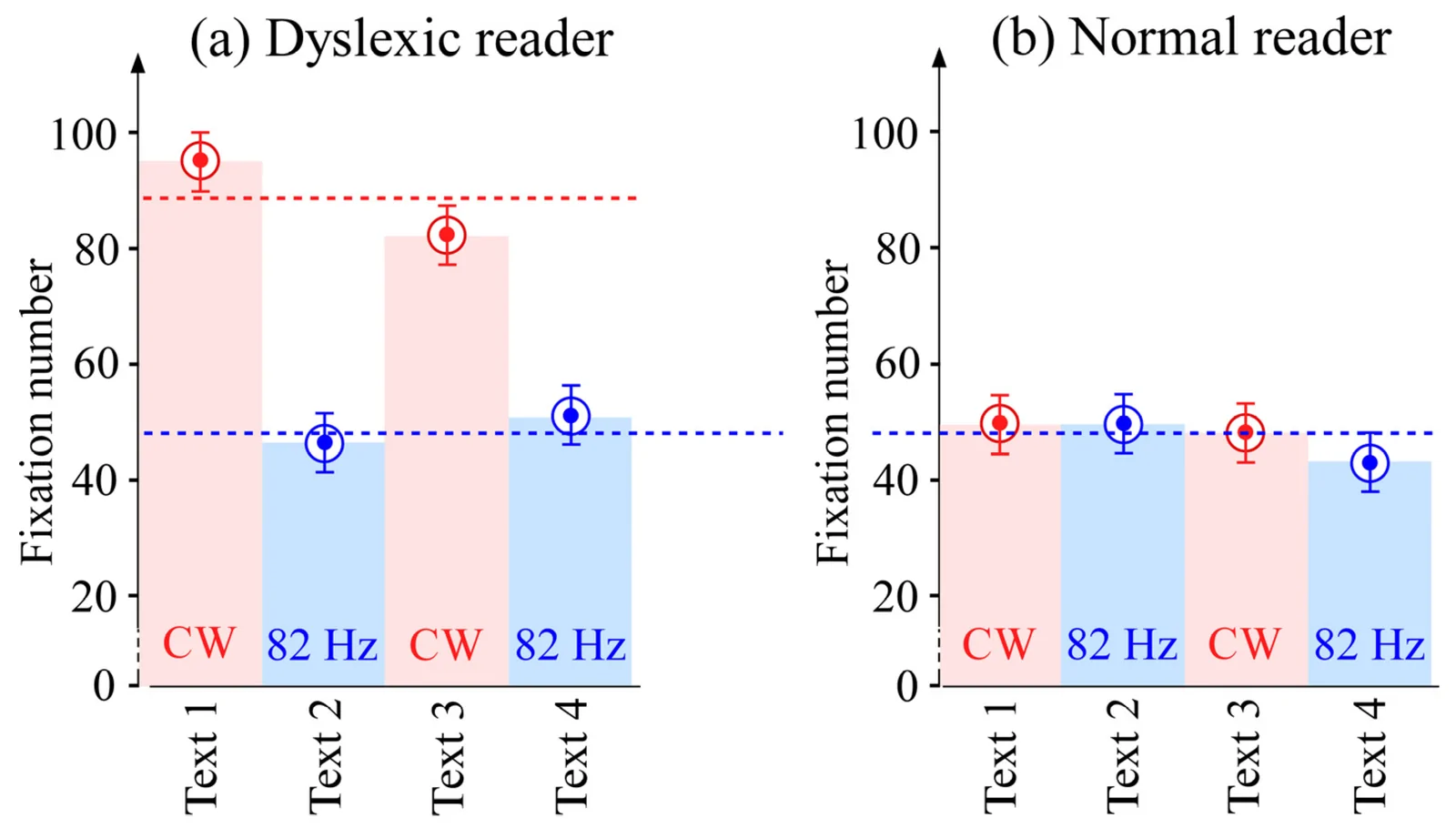
Number of fixations for four different texts. (**a**) Results for the dyslexic reader in the two regimes. (**b**) Results for the normal reader in the two regimes. The red zones correspond to the continuous regime, and the blue zones correspond to the pulsed regime.

Repeating the experiment for four different texts yielded the results schematized in Figure 5. The error bars represent the estimated errors. The errors were estimated in a way similar to that used in [11], taking into account the inaccuracies of the recording. For the dyslexic reader (Figure 5a), the number of fixations was reduced by a factor of about 1.8 in the pulsed regime. Without the internal visual crowding, the level of the reader with dyslexia improves to that of a normal reader (Figure 5b). 

**Figure 6:**
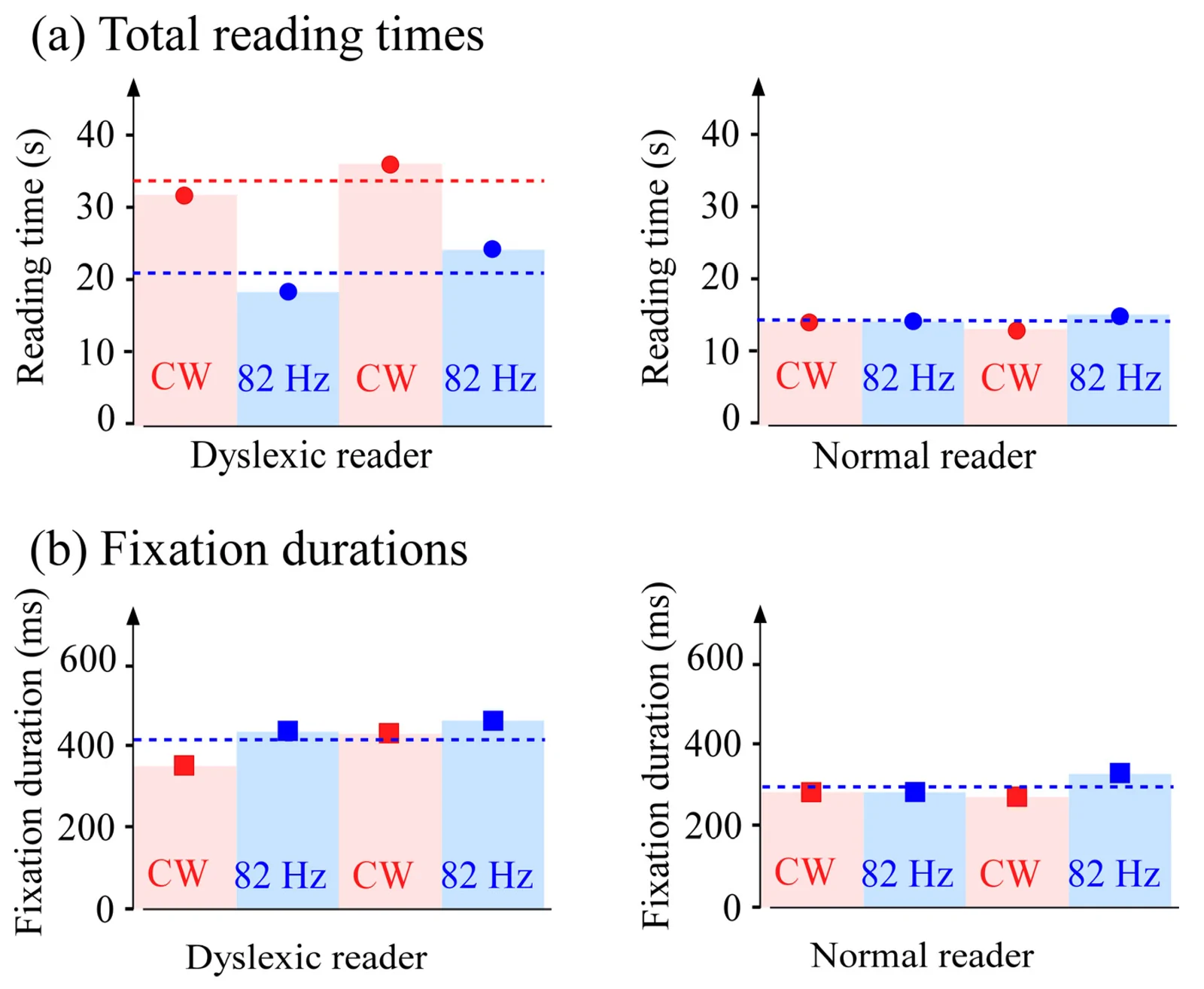
Comparative durations for the dyslexic and control readers. (**a**) Total duration for four texts for the two readers in the two lighting regimes. For the dyslexic reader, at 82 Hz, the total duration is reduced by a factor 1.6. (**b**) Invariance of the fixation durations for the two readers in the two lighting regimes; the fixation durations remain longer for the dyslexic reader. The red zones correspond to the continuous regime and the blue zones correspond to the pulsed regime.

The total reading times of the two readers are shown in Figure 6a. While the reading time is invariant for the normal reader, the total time is reduced by a factor of about 1.6 in the pulsed regime for the dyslexic reader but remains longer than that of the normal reader. The fixation durations are shown in Figure 6b. For both readers, the duration times are quasi invariant, but the fixation duration remains longer for the dyslexic reader by about 30%.

## 4. Discussion

Our eye tracking experiment confirms that the eye movements of the reader with dyslexia are different from those of a normal reader (Figure 4). In particular, the dyslexic reader makes more and longer fixations (about twice as many as a normal reader) and has longer reading times. Such observations have been made in different languages [12,13,15,30]. However, the causal relationship remains the subject of debate [44]. A lack of asymmetry between the Maxwell centroids of the two foveas has been shown to induce internal visual crowding in many readers with dyslexia [41,50], together with postural instabilities [51]. Indeed, the retinal images of the two eyes are excessively similar, inducing excessively similar retinoptic maps on layer 4 of the primary cortex, where the ganglion cells of the retinas reach the cortex. The interhemispheric projections through the corpus callosum between the excessively similar neuronal topographies in the two hemispheres are stronger than those for a normal reader with an asymmetry. When this is the case, the symmetric projections lead to superposed primary and mirror images and are perceived by the dyslexic reader not only for letters but also for words, as shown in Figure 2b. Internal visual crowding was absent for the normal reader (Figure 2c), and internal visual crowding cannot be geometrically weakened by spacing effects like external crowding, which can also induce impairments in reading [34].

In contrast, internal visual crowding has been shown to be erasable using the Hebbian mechanisms [43] at the synapses of the primary cortex [41]. Indeed, as the projected mirror images have to travel through the corpus callosum, they are delayed by about 10 milliseconds, corresponding to the transit time between the two hemispheres [52]. Pulse-width modulation of the light of the computer screen at frequencies beyond the visible flicker allows the mirror images to be weakened, restoring a single primary image similar to that perceived by a normal reader. When the modulation frequency is optimized for the dyslexic reader (here at 82 Hz), the internal visual crowding is completely erased and the number of fixations is immediately reduced to the normal reader regime. The responses of the normal reader remain invariant whatever the light regime as there is no internal visual crowding (see Figure 5 and Figure 6). The causality relationship between the internal visual crowding and the number of fixations is objectively established with an immediate and quantitative effect.

To conclude, the lack of asymmetry between the two Maxwell centroids in a reader with dyslexia, which results in a lack of ocular dominance and the existence of internal visual crowding, leads to a greater number of fixations and longer reading durations. Indeed, the excessively strong interhemispheric projections generally induce either perceived extra mirror or duplicated images [50], which make reading difficult by increasing linguistic and cognitive processing demands, in contrast to other visual tasks. Using the Hebbian mechanisms at the synapses in the primary cortex (activated by an optimized pulsed light regime from an electronically modified computer screen), the problematic internal crowding can be reduced, and excessive fixations controlled to regain the level of reading demonstrated by normal readers. Although we have compared the results for only one dyslexic reader with those of a normal reader in this paper, since the method uses common tracking features, we hope that the results will be confirmed by other research groups using larger samples. Other potential brain correlates can probably be observed, but ocularly tracking the fixations provides an immediate, precise, and objective quantification of the reduction in the number of fixations in reading and suggests a causality relationship between the reading deficit and internal visual crowding. Moreover, the reduction in the excess of fixations in reading observed here could be used as a diagnostic tool for dyslexia.

## 5. Patents

A patent has been filed by the University of Rennes for the modified computer screen.

## Data Availability

This article has no additional data.
